# Biofilm formation of mucosa-associated methanoarchaeal strains

**DOI:** 10.3389/fmicb.2014.00353

**Published:** 2014-07-08

**Authors:** Corinna Bang, Claudia Ehlers, Alvaro Orell, Daniela Prasse, Marlene Spinner, Stanislav N. Gorb, Sonja-Verena Albers, Ruth A. Schmitz

**Affiliations:** ^1^Institute for General Microbiology, University of KielKiel, Germany; ^2^Molecular Biology of Archaea, Max Planck Institute for Terrestrial MicrobiologyMarburg, Germany; ^3^Molecular Microbiology of Extremophiles Research Group, Centre for Genomics and Bioinformatics, Faculty of Sciences, Universidad MayorSantiago, Chile; ^4^Functional Morphology and Biomechanics, Zoological Institute, University of KielKiel, Germany

**Keywords:** biofilms, methanoarchaea, human gut, microbiota

## Abstract

Although in nature most microorganisms are known to occur predominantly in consortia or biofilms, data on archaeal biofilm formation are in general scarce. Here, the ability of three methanoarchaeal strains, *Methanobrevibacter smithii* and *Methanosphaera stadtmanae*, which form part of the human gut microbiota, and the *Methanosarcina mazei* strain Gö1 to grow on different surfaces and form biofilms was investigated. All three strains adhered to the substrate mica and grew predominantly as bilayers on its surface as demonstrated by confocal laser scanning microscopy analyses, though the formation of multi-layered biofilms of *Methanosphaera stadtmanae* and *Methanobrevibacter smithii* was observed as well. Stable biofilm formation was further confirmed by scanning electron microscopy analysis. *Methanosarcina mazei* and *Methanobrevibacter smithii* also formed multi-layered biofilms in uncoated plastic μ-dishes^TM^, which were very similar in morphology and reached a height of up to 40 μm. In contrast, biofilms formed by *Methanosphaera stadtmanae* reached only a height of 2 μm. Staining with the two lectins ConA and IB4 indicated that all three strains produced relatively low amounts of extracellular polysaccharides most likely containing glucose, mannose, and galactose. Taken together, this study provides the first evidence that methanoarchaea can develop and form biofilms on different substrates and thus, will contribute to our knowledge on the appearance and physiological role of *Methanobrevibacter smithii* and *Methanosphaera stadtmanae* in the human intestine.

## INTRODUCTION

Growth of microorganisms as complex microbial communities is the predominant lifestyle in nature and has been shown to occur on a wide variety of surfaces including living tissues (Donlan, 2002). Although the human gut harbors trillions of microorganisms forming a complex ecological community (Whitman et al., 1998; Hopkins et al., 2001; Macpherson and Harris, 2004; Abreu et al., 2005; Ley et al., 2006; O’Hara and Shanahan, 2006; Artis, 2008; Lozupone et al., 2012), the existence and significance of mucosa-associated biofilms was not considered for many years (Dongari-Bagtzoglou, 2008). However, during the last decade, the increasing numbers of studies dealing with the overall microbial diversity in the human gut have demonstrated bacterial biofilm formation on the mucus itself or the epithelial surface (Macfarlane and Dillon, 2007; Macfarlane et al., 2011). In this regard, the biofilm development on mucosal surfaces was shown to depend not only on environmental and nutritional factors but also on the host defense mechanisms (Macfarlane and Dillon, 2007). Particularly in patients suffering from inflammatory bowel diseases (IBD) the density and composition of mucosal biofilms has been shown to alter significantly when compared to healthy controls (Swidsinski et al., 2005). Biofilm formation on human mucosa surfaces are so-called “mucosal biofilms” involving microbial adhesion to the mucosa with subsequent cell-to-cell adhesion leading to multicellular structure formation (Post et al., 2004; Dongari-Bagtzoglou, 2008). Structurally, members of those biofilms are embedded in a matrix of extracellular polymeric substances (EPS) that mediates protective functions as well as nutrient supply and enables communication between biofilm forming microorganisms (Flemming and Wingender, 2010). In addition, biofilm-associated microorganisms are phenotypically different from their planktonic counterpart, as indicated by the finding that large suites of genes are differentially transcribed (An and Parsek, 2007). Whereas environmental biofilms are mostly composed of various microbial species, medically relevant biofilms on epithelial tissues (such as the lung, the gut and the oral cavity) that are associated with infectious diseases are often composed of just a few species (Donlan, 2002). In this respect, diversity in mucosal biofilms was also found to be low, when compared to the overall microbial diversity in the human gut (Swidsinski et al., 2005; Dongari-Bagtzoglou, 2008). Studies of mucosal biofilms are mainly exclusively focused on bacterial species, though several members of the archaeal domain have been identified to be stable components of the complex microbial community in the human gut (Whitman et al., 1998; O’Hara and Shanahan, 2006; Hill and Artis, 2010). In particular, the methanoarchaea *Methanobrevibacter smithii* and *Methanosphaera stadtmanae* are known to be part of the human gut microbiota (Miller et al., 1982, 1984; Lovley et al., 1984; Miller and Wolin, 1985; Weaver et al., 1986; Backhed et al., 2005; Eckburg et al., 2005; Levitt et al., 2006; Dridi et al., 2009). Notably, *Methanobrevibacter smithii* has been shown to inhabit nearly every human individual gut ecosystem, whereas *Methanosphaera stadtmanae* was found in 30% of individuals (Dridi et al., 2009; Dridi, 2012). Both strains, *Methanobrevibacter smithii* and *Methanosphaera stadtmanae*, have been shown to be involved in fermentation processes by converting bacterial fermentation products like hydrogen, organic acids (e.g., formate, acetate), and carbon dioxide, to methane (Miller et al., 1984; Samuel and Gordon, 2006; Samuel et al., 2007). Apart from that, the knowledge on further functions of *Methanobrevibacter smithii* and *Methanosphaera stadtmanae* in the human intestinal ecosystem is still limited, though *Methanobrevibacter smithii*’s role in the development of adiposity was proposed in several studies (Samuel et al., 2008; Mathur et al., 2013). Very recently, an influence of those predominating methanoarchaeal strains on the immunomodulation within the human intestine was obtained (Bang et al., 2014). In addition, *Methanobrevibacter oralis*, which is a close relative of *Methanobrevibacter smithii*, was anticipated to play a role in the manifestation of periodontal disease and meanwhile its prevalence was shown to be increased in patients suffering from chronic periodontitis (Kulik et al., 2001; Vianna et al., 2006; Ashok et al., 2013). In general, these findings argue that the impact of (methano)archaea on human’s health and disease might have been underestimated until now.

With respect to the identified syntrophic interactions between methanoarchaea and bacterial gut inhabitants (Samuel and Gordon, 2006; Samuel et al., 2007), it appears most likely that methanoarchaeal strains occur as biofilms within the human intestine together with gut bacteria such as *Bacteroides* species (Swidsinski et al., 2005). However, information on archaeal biofilm formation is in general rare and only a few examples are reported, which are reviewed in Fröls (2013) and Orell et al. (2013). On the other hand, it is known that the methanoarchaeal strain *Methanosarcina mazei* easily forms cellular aggregates in the presence of environmental stressors (Mayerhofer et al., 1992). Thus, understanding how methanoarchaea interact with gut bacteria and the mucosa itself potentially by forming biofilms is crucial for upcoming studies dealing with the immunomodulatory role of those microorganisms. Consequently, the aim of this study was to evaluate the general ability of the methanoarchaeal gut inhabitants *Methanobrevibacter smithii* and *Methanosphaera stadtmanae* to form biofilms on two different substrates as well as to examine structural characteristics of these biofilms, in particular in comparison with a methanoarchaeon originally isolated from sewage sludge, *Methanosarcina mazei* strain Gö1.

## MATERIALS AND METHODS

### STRAINS AND GROWTH CONDITIONS

*Methanosarcina mazei* strain Gö1 (DSM 3647), *Methanosphaera stadtmanae* (DSM 3091) and *Methanobrevibacter smithii* (DSM 861) were obtained from the Deutsche Sammlung von Mikroorganismen und Zellkulturen (DSMZ, Braunschweig, Germany). *Methanosarcina mazei* strain Gö1 was grown in minimal medium under strict anaerobic conditions as described earlier (Ehlers et al., 2002; Bang et al., 2012). *Methanosphaera stadtmanae* was grown in medium 322 (according to the DSMZ, www.dsmz.de) and *Methanobrevibacter smithii* in medium 119 (according to the DSMZ, http://www.dsmz.de) both containing 10% rumen fluid. The reductants Na_2_S (1.25 mM) and cysteine (2.5 mM) were added after autoclaving of media and 1.5 atm H_2_/CO_2_ (80/20 vol/vol) was used as a gas phase. Medium for *Methanosphaera stadtmanae* was further complemented with 150 mM methanol prior inoculation. To prevent bacterial contamination, the medium for all strains was in general supplemented with 100 μg/ml ampicillin.

### GROWTH ON MICA

For initial adherence experiments of the methanoarchaeal strains, mica plates (Baltic Präparation, Niesgrau, Germany) with an edge length of 0.5 cm were used. Those mica pieces were transferred into hungate tubes, autoclaved, and placed into an anaerobic chamber with an atmosphere of N_2_/CO_2_/H_2_ (78/20/2 vol/vol/vol), which was constantly circulated through a 0.3 μm filter system (Coy Laboratory Products Inc., MI, USA) to ensure anaerobic and semi-sterile conditions. At least 24 h later, 3 ml of reduced and complemented media were filled in the prepared hungate tubes and 1 × 10^7^ cells of the respective methanoarchaeal preculture during its exponential growth phase were added. Those preparations were vertically incubated and samples were taken after 48, 72, and 96 h. Samples for microscopic analysis were fixed with 2% glutaraldehyde (Sigma-Aldrich Biochemie GmbH, Hamburg, Germany, Number G5882), which was directly added to the hungate tubes for at least 4 h at 4^∘^C prior washing in minimal medium and microscopic examination at 1000× magnification using an Axio Lab microscope (Carl Zeiss MicroImaging GmbH, Jena, Germany) supplied with a digital camera (AxioCam Mr5, Carl Zeiss MicroImaging GmbH). Phase-contrast micrographs were captured using the digital image analysis software AxioVision Rel. 4.7.1 (Carl Zeiss MicroImaging GmbH). In addition, fixed samples after 48 h of growth were visualized by using the autofluorescence of glutaraldehyde in a TCS-SP5 confocal laser scanning microscope (Leica, Bensheim, Germany) at an excitation wavelength of 520 nm and an emission wavelength of 540 nm. Obtained image data were edited by using the IMARIS software package (Bitplane AG, Zürich, Switzerland).

### SCANNING ELECTRON MICROSCOPY (SEM)

After growing periods of 48 h (*Methanosarcina mazei, Methanosphaera stadtmanae*) or 72 h (*Methanobrevibacter smithii*) cultures were prepared as described above and mica plates were fixed on the aluminum stubs with double-sided carbon conductive tapes (Plano, Wetzlar, Germany). Subsequently, samples were air dried in a desiccator with silica gel (Merck KGaA, Darmstadt, Germany) for a period of 72 h. After coating with a 10 nm thick layer of gold-palladium in a sputter coater (Leica EM SCD500, Leica Microsystems GmbH, Wetzlar, Germany), samples were examined in SEM Hitachi S-4800 (Hitachi High-Technologies Corp., Tokyo, Japan) at an accelerating voltage of 3 kV.

### CONFOCAL LASER SCANNING MICROSCOPY (CLSM)

For CLSM images, the cells were grown for 72 h in uncoated plastic dishes^TM^ (μ-Dishes^TM^, 35 mm high; Ibidi, Martinsried, Germany). Prior to confocal microscopy, the liquid supernatant of the biofilm, with the planktonic cells, was removed and 2 ml fresh medium was added. Images were recorded on an inverted TCS-SP5 confocal microscope (Leica). DAPI (4,6-diamidino-2-phenylindole), dissolved in water to 300 μg/ml, was used to visualize the cells of the biofilm. For this reason, 7 μl of the DAPI stock solution in 2 ml fresh medium were added to the biofilm, incubated at room temperature for at least 10 min and subsequently washed twice with 2 ml fresh medium. Images were taken at an excitation wavelength of 345 nm and an emission wavelength of 455 nm. Fluorescently labeled lectins were employed to visualize the EPS (extracellular polymeric substances) of the biofilms. Prior addition of lectins to the biofilm, fluorescein-conjugated concanavalin A (ConA; 5 mg/ml; Life Technologies GmbH, Darmstadt, Germany), which binds to α-mannopyranosyl and α-glucopyranosyl residues, was dissolved in 20 mM sodium bicarbonate (pH 8.0) to a final concentration of 10 mg/ml. Fluorescein-conjugated ConA has an excitation wavelength of 494 nm and an emission wavelength of 518 nm. Alexa FluorH594-conjugated IB4, specific for α-D-galactosyl residues (isolectin GS-IB4 from *Griffonia simplicifolia* 1 mg/ml; Life Technologies GmbH) was dissolved in 100 mM Tris-HCl pH 7.4 and 0.5 mM CaCl_2_ to a final concentration of 8 mg/ml. The Alexa Fluor-conjugated lectin, which has an excitation wavelength of 591 nm and an emission wavelength of 618 nm, was used in concert with ConA. The lectin–biofilm mixtures were incubated at room temperature for 20–30 min in the absence of light. After incubation, the biofilm was washed with fresh media to remove excess label and images were taken by CSLM. Image data were processed by using the IMARIS software package (Bitplane AG).

### DETERMINATION OF SURFACE COVERAGE

To evaluate cell surface coverage of the biofilms, pictures of the bottom layer were taken using a differential interference contrast (DIC) objective. Twelve images at different microscopy fields were recorded. By using Adobe Photoshop CS2 software DIC pictures were converted into black/white in order to calculate number of pixels/area thus representing the percentage surface coverage. Cell surface coverage determinations were performed in three biological replicates.

## RESULTS

The aim of this study was to examine the general ability of several methanoarchaeal strains to form biofilms and to evaluate potential differences between the human gut inhabitants *Methanobrevibacter smithii* and *Methanosphaera stadtmanae* as well as *Methanosarcina mazei* strain Gö1, a member of the Methanosarcinales inhabiting various anoxic environments (Deppenmeier et al., 2002; Chaban et al., 2006).

**FIGURE 1:**
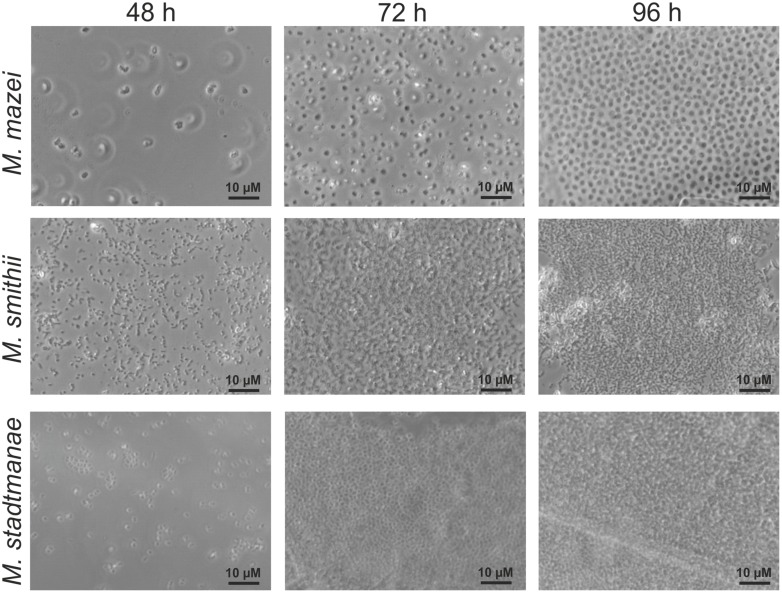
**Growth of different methanoarchaea on mica.** *Methanosarcina mazei*, *Methanobrevibacter smithii*, and *Methanosphaera stadtmanae* were grown in 3 ml standard medium under an N_2_/CO_2_ atmosphere for *Methanosarcina mazei* or an H_2_/CO_2_ gas phase for *Methanobrevibacter smithii* and *Methanosphaera stadtmanae*; the cultures were supplemented with 1–2 pieces of mica. Growth on mica of all three strains was monitored by phase-contrast microscopy at defined time points of 48, 72, and 96 h.

**FIGURE 2:**
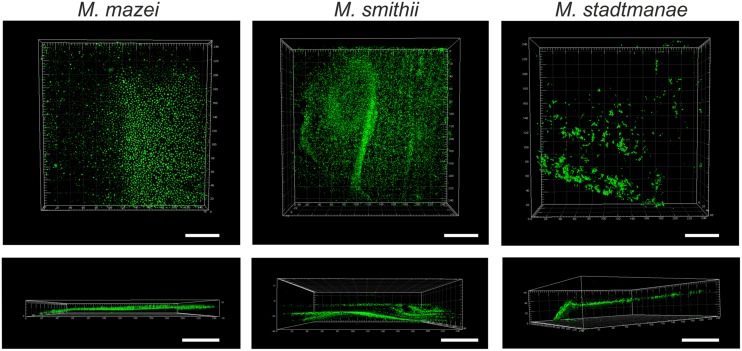
**Growth of methanogens on mica examined by confocal laser scanning microscopy.** *Methanosarcina mazei*, *Methanobrevibacter smithii,* and *Methanosphaera stadtmanae* were grown on mica in hungate tubes with 3 ml of the respective medium. After 48 h of growth, cells were fixed to mica by 2% glutaraldehyde. The autofluorescence of glutaraldehyde was used for CLSM pictures at a wavelength of 520 nm. The scale bar is 50 μm.

**FIGURE 3:**
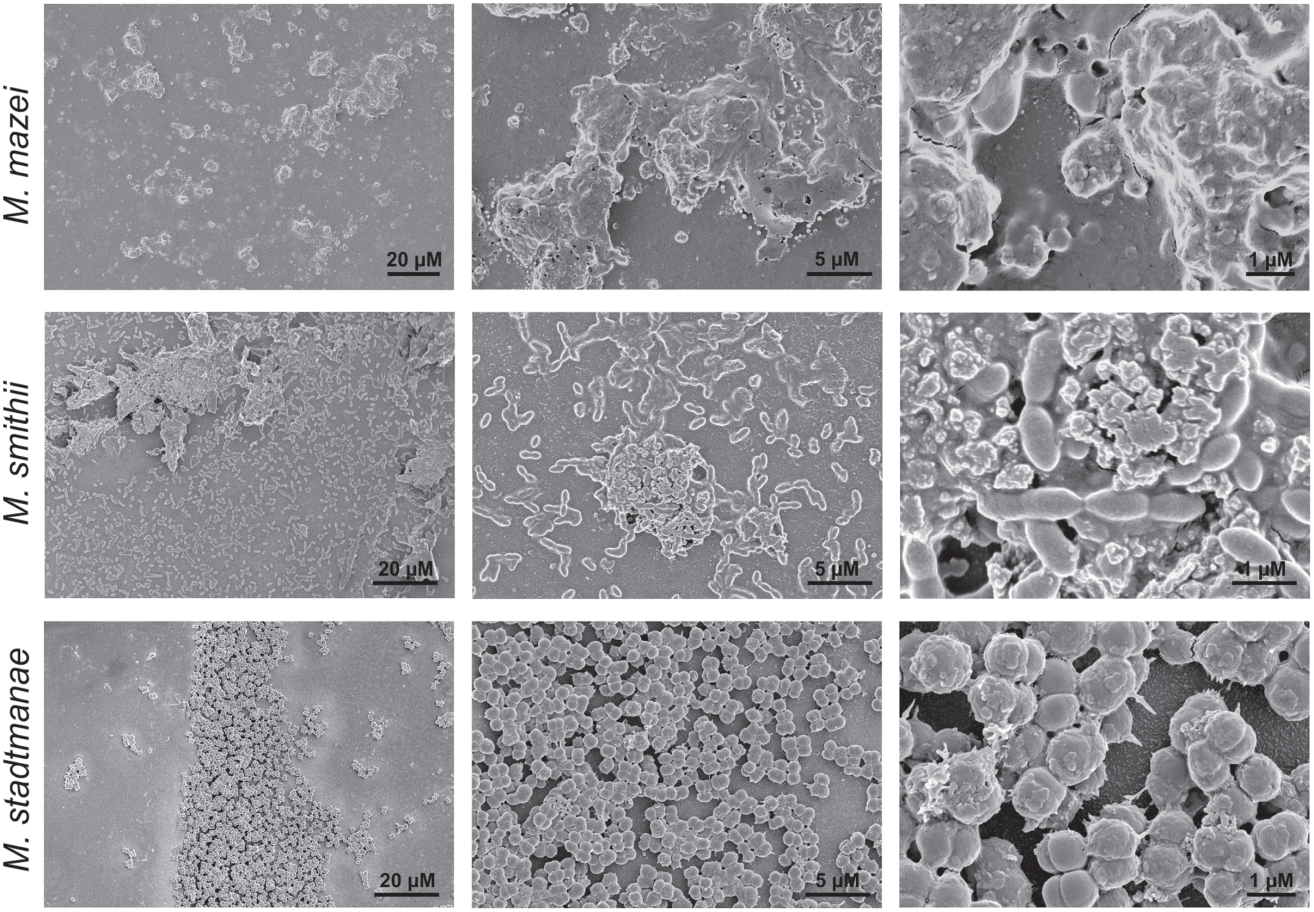
**Growth of methanogens on mica examined by SEM.** *Methanosarcina mazei*, *Methanobrevibacter smithii,* and *Methanosphaera stadtmanae* were grown on mica in hungate tubes with 3 ml of the respective medium. After 48 h (*Methanosarcina mazei* and *Methanosphaera stadtmanae*) and 72 h (*Methanobrevibacter smithii*) of growth, cells were fixed to mica by 2% glutaraldehyde. Images are representative for the respective sample.

Since no information was available on biofilm formation of methanoarchaeal gut inhabitants, initially static growth of *Methanobrevibacter smithii* and *Methanosphaera stadtmanae* as well as of *Methanosarcina mazei* strain Gö1 on mica plates was investigated. For this purpose, methanoarchaeal strains were grown for varying time periods in strain-specific media containing small pieces of mica plates. These preparations were fixed with 2% glutaraldehyde and washed prior to the subsequent analysis. Phase-contrast microscopic examination of these mica plates after 48, 72, and 96 h revealed growth on mica for all three strains with increasing cell numbers during the time course (**Figure 1**). However, differences in the phenotype of the strains were observed during biofilm development. On the one hand, even after 96 h a precise space between the high numbers of attached *Methanosarcina mazei* cells resulting in no direct cell-to-cell contact was observed, which might potentially be coordinated by pili or EPS components. On the other hand, cells of *Methanobrevibacter smithii* and *Methanosphaera stadtmanae* strongly formed aggregates attached to the surface with increasing cell numbers (**Figure 1**). In addition, all three strains appeared to form predominantly bilayer biofilms (**Figures 1–3**), although multi-layered growth was occasionally observed for *Methanobrevibacter smithii* and *Methanosphaera stadtmanae* (**Figure 3**).

Confocal laser scanning microscopy was used to further visualize biofilm formation by the methanoarchaeal strains after 48 h on the prepared mica plates. The autofluorescence of glutaraldehyde enabled visualization of methanoarchaeal cell growth on the surface of mica plates by applying the respective wavelength (520 nm). This analysis revealed widespread adhesion of *Methanosarcina mazei* and *Methanobrevibacter smithii* cells over the surface of mica plates, whereas only small areas were shown to be overgrown by *Methanosphaera stadtmanae* (**Figure 2**). Since comparable initial cell numbers of all strains were used as inoculum, these results demonstrated that *Methanobrevibacter smithii* and *Methanosarcina mazei* adhered better to the smooth surface of the mica when compared to *Methanosphaera stadtmanae*.

Further morphological characteristics of the methanoarchaeal biofilms were analyzed by using SEM. Cell-to-cell adhesion as well as adhesion to the mica surface could be demonstrated using this method for *Methanobrevibacter smithii* and *Methanosphaera stadtmanae* (**Figure 3**). In addition, secretion of potential extracellular polymeric substances (EPS) by all tested strains was observed (**Figure 3**). The secretion of this potential EPS by *Methanobrevibacter smithii* and *Methanosphaera stadtmanae* rose with increasing attached cell numbers; however, the highest production of potential EPS was detected for *Methanosarcina mazei*. Probably due to the air dry conditions, *Methanosarcina mazei* cells lost their integrity and thus, in SEM analysis of *Methanosarcina mazei* no single cells were found (**Figure 3** *Methanosarcina mazei*). Since difficulties during the preparation procedures of *Methanosarcina mazei* for electron microscopy analyses were already observed during an earlier study (Bang et al., 2012), SEM analysis only indicated the general ability of *Methanosarcina mazei* to form biofilms on mica.

**FIGURE 4:**
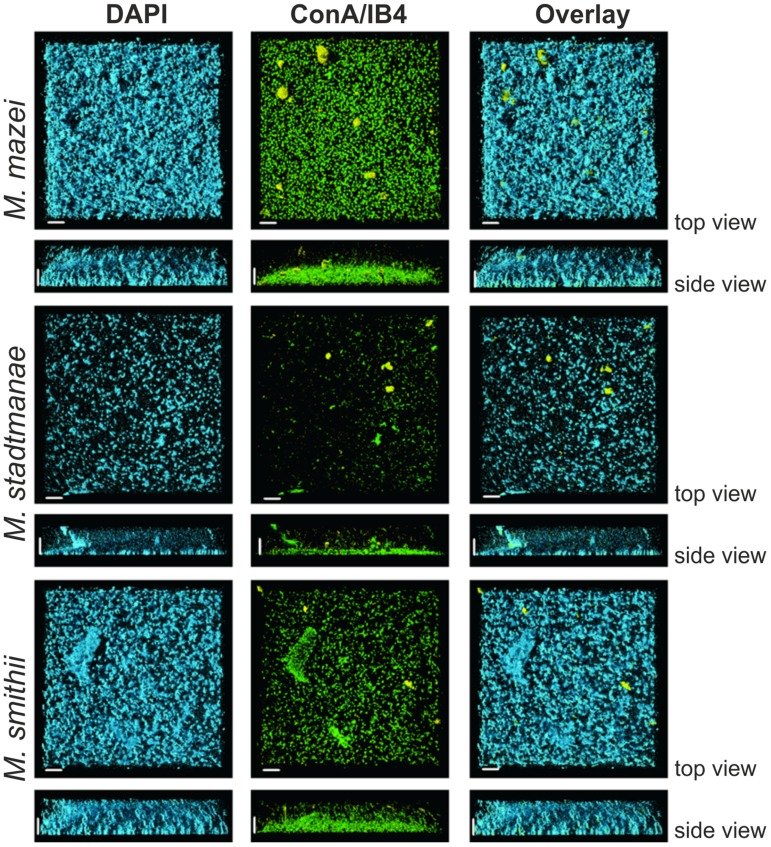
**Structures of static biofilms formed by *Methanosarcina mazei*, *Methanosphaera stadtmanae,* and* Methanobrevibacter smithii*.** Cells were grown in 4 ml standard medium in μ-dishes^TM^ under the respective gas atmosphere. After 72 h of growth, the biofilms were treated with DAPI (blue channel), ConA (green channel) and IB4 (yellow channel) and visualized by CLSM; single channels and overlays of the images are displayed. Both top view (upper lane) and side view (lower lane) of the biofilms are shown. The scale bar is 20 μm.

For a more detailed analysis of the biofilm formation, *Methanosarcina mazei*, *Methanobrevibacter smithii*, and *Methanosphaera stadtmanae* were incubated in strain-specific medium under static conditions in uncoated plastic μ-dishes^TM^ for 72 h. Subsequently, the biofilms formed were analyzed by CLSM and DAPI was used to visualize the cells. Structurally, this method revealed that *Methanosarcina mazei* and *Methanobrevibacter smithii* formed multi-layered biofilms being very similar in respect to their morphology and height (up to 40 μm; **Figure 4**, DAPI). However, biofilms formed by *Methanobrevibacter smithii* appeared to be denser and more compacted when compared to *Methanosarcina mazei*. In contrast to *Methanosarcina mazei* and *Methanobrevibacter smithii*, biofilms formed by *Methanosphaera stadtmanae* developed only to a height of 2 μm, with occasional tower-like structures unevenly distributed on the surface (**Figure 4**, left panel, DAPI).

In order to confirm the observed production of potential EPS by the methanoarchaeal biofilms (visible in **Figure 3**), these sessile communities were additionally stained using two different fluorescently labeled lectins, ConA and IB4. A strong ConA signal was observed in biofilms formed by all three strains, indicating the presence of glucose and/or mannose residues. However, the ConA signal (**Figure 4**, green signal) closely co-localized with the DAPI stained cells (**Figure 4**, blue signal). On the contrary, the IB4 signal (**Figure 4**, yellow signal), which is specific for α-galactosyl sugar residues, was only detected in very few clusters in all three biofilms and appeared not to be directly co-localized with cells.

**FIGURE 5:**
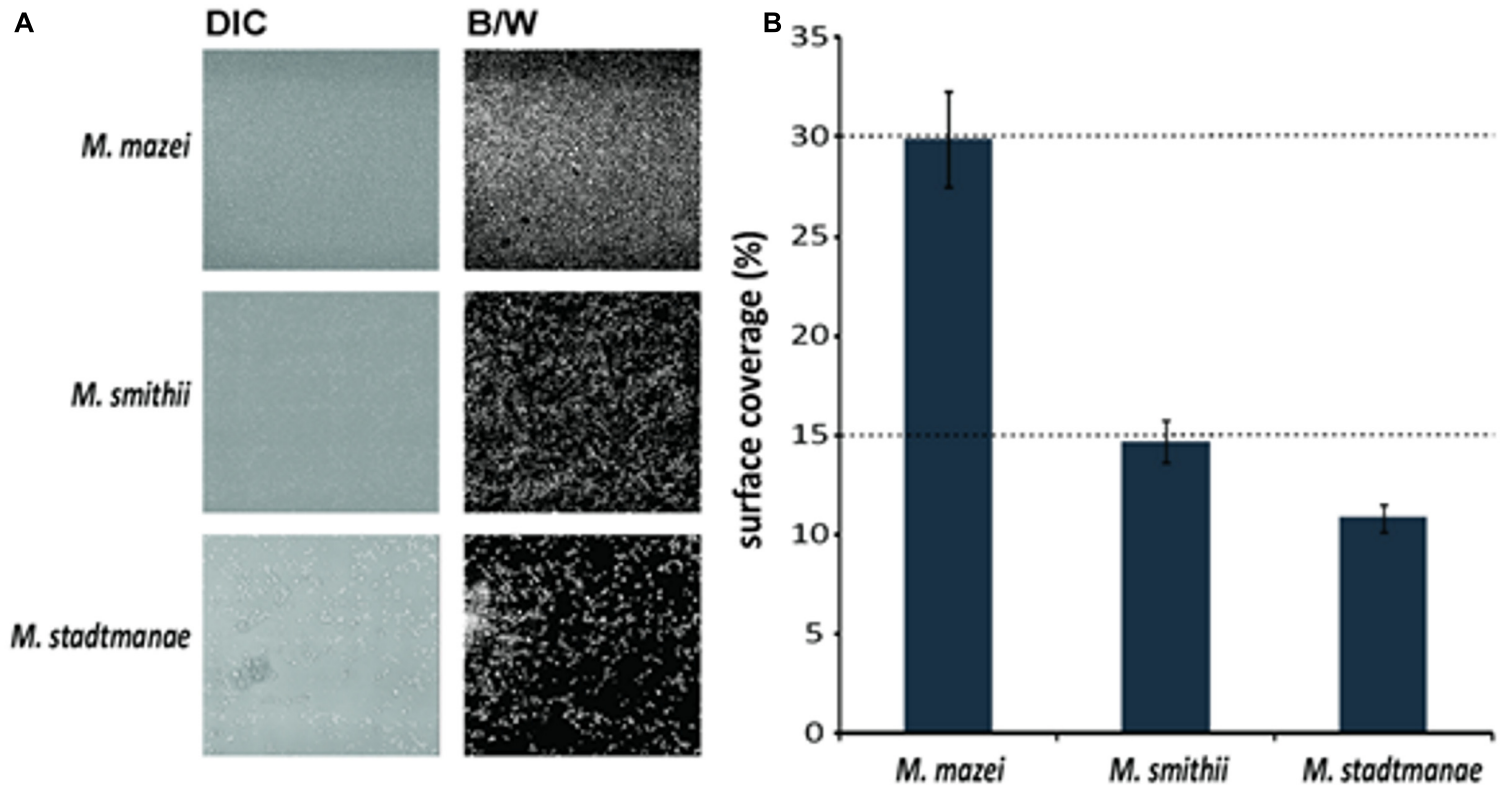
**Analysis of the surface coverage of biofilms formed by *Methanosarcina mazei*, *Methanosphaera stadtmanae,* and* Methanobrevibacter smithii.*** Differential interference contrast (DIC) pictures (**A**, left panel) were taken from the bottom layer of static biofilms and converted into black/white (B/W; **A**, right panel) to calculate the surface coverage. The ratio of B/W pixels was determined and used to obtain the surface coverage **(B)**. The mean and standard deviations of three biological replicates are shown.

The bottom layers of the static biofilms formed by *Methanosarcina mazei*, *Methanosphaera stadtmanae*, and* Methanobrevibacter smithii* were imaged in order to calculate the respective surface coverage of the biofilms. This analysis revealed 50% higher coverage of the surface at the bottom of the μ-dish in the *Methanosarcina mazei* biofilm, when compared to the *Methanobrevibacter smithii* biofilm (**Figure 5**). Moreover, the surface coverage of the bottom layer of biofilms formed by *Methanosphaera stadtmanae* was found to be only 30% of the one from *Methanosarcina mazei* and about 70% of the *Methanobrevibacter smithii* biofilm (**Figure 5**). However, it cannot completely ruled out that the surface coverage analysis of *Methanosarcina mazei* was affected by the potential EPS structures surrounding cells of *Methanosarcina mazei*, which were observed during SEM-analysis (**Figure 3**).

## DISCUSSION

Although the knowledge on the functional importance of mucosal biofilms clearly increased in the last decade, the diversity and characteristics of microbial communities associated with the human gut mucosa are still poorly understood (Dongari-Bagtzoglou, 2008). In addition, most studies dealing with the development and composition of human gut mucosal biofilms did only involve bacterial or fungal species (Swidsinski et al., 2005; Macfarlane and Dillon, 2007; Macfarlane et al., 2011). Thus, to our knowledge, this is the first report demonstrating biofilm formation of methanogenic archaea that frequently inhabit the human gut. By assessing static growth on different surfaces (mica and uncoated plastic μ-dishes^TM^) we showed that the studied methanoarchaeal strains, *Methanosarcina mazei*, *Methanobrevibacter smithii,* and *Methanosphaera stadtmanae*, form biofilms with distinctive features. As it has been shown for other few archaeal species that form biofilms such as *Sulfolobus* spp. (Koerdt et al., 2010, 2011), the SM1 Euryarchaeon (Probst et al., 2013), several haloarchaeal strains (Fröls et al., 2012) and *Pyrococcus furiosus* as well as *Methanopyrus kandleri* (Schopf et al., 2008), each studied strain showed strain-specific characteristics during biofilm formation that were observed by using various microscopic techniques such as CLSM and SEM. In particular, significant differences in biofilm forming capabilities of the human gut inhabitants *Methanosphaera stadtmanae* and *Methanobrevibacter smithii* were observed. In μ-dishes^TM^, *Methanobrevibacter smithii* biofilms reached heights up to 40 μm, whereas *Methanosphaera stadtmanae* biofilms grew only up to a height of 2 μm. However, surface coverage of *Methanosphaera stadtmanae* (∼11%) was found to be almost similar to that obtained for *Methanobrevibacter smithii* (∼15%). Regarding to this, it has been shown in earlier studies that biofilm thickness and density increase with the number of participating microorganisms within the community (Costerton et al., 1995; Donlan, 2002). Thus, biofilm-forming communities consisting of both, bacteria and archaea, may reach significantly higher heights and surface coverage as has been shown for various environmental biofilms (Orell et al., 2013). Furthermore, it has been demonstrated that bacterial human mucosal biofilm formation is favored in fluid flow or tissue motility such as the human gut (Tolker-Nielsen et al., 2000; Donlan, 2002; Dongari-Bagtzoglou, 2008). Hence, the determined static biofilm formation of methanoarchaeal strains might underestimate their overall* in vivo* ability to form mucosal biofilms within the human gut. Interestingly, the observed biofilm forming capabilities of the tested methanoarchaeal strains differed within the two used systems. In particular, *Methanosphaera stadtmanae*’s biofilm formation on mica plates appeared more pronounced when compared to the growth in μ-dishes^TM^. While the used mica plates are very smooth and hydrophilic, the surface of uncoated μ- dishes^TM^ is more roughened and hydrophobic. Thus, surface properties are likely to influence the overall ability of methanoarchaeal strains to form biofilms.

By using several lectins, only very low amounts of EPS were detected in these methanoarchaeal biofilms (**Figure 4**). This observation might be due to the fact that the tested lectins did not exhibit the specificity needed to detect the secreted polysaccharides, since SEM analysis revealed high production of EPS for at least *Methanosphaera stadtmanae* and *Methanobrevibacter smithii*. The tested lectin ConA mainly recognizes glucose and mannose residues, which form major components of EPS. However, the ConA signal was mainly co-localized with the DAPI stained cells; thus implying that the stained compound did not correspond to secreted exopolysaccharides, but most likely to the *N*-glycans that cover the outmost sheath of proteins or heteropolysaccharides surrounding the methanoarchaeal cell surface (König, 1988, 2010; Kandler and König, 1998). In addition, the lectin IB4, specific for α-galactosyl residues, was rarely observed in all three biofilms. In this respect, further analysis is required to determine carbohydrate moieties of secreted EPS by methanoarchaeal strains. On the other hand, high amounts of extracellular DNA (eDNA) have been observed in archaeal biofilms during earlier studies, particularly located in regions of sessile cell aggregates (Fröls et al., 2008; Koerdt et al., 2010; Orell et al., 2013). Hence, future studies should also include examination of eDNA with an membrane-impermeable DNA-intercalating dye as well as detection of secretion proteins.

SEM analysis in this study revealed not only adhesion of methanoarchaeal strains to the smooth mica surface, but also strong cell-to-cell adhesion of at least *Methanosphaera stadtmanae* and *Methanobrevibacter smithii* during biofilm formation. The functional role of bacterial type-IV-pili-like structures and non-type-IV-pili-like structures involved by various archaeal species in biofilm formation has been confirmed in earlier studies (Fröls et al., 2008; Henche et al., 2012). However, the genomes of *Methanosphaera stadtmanae* and *Methanobrevibacter smithii* lack coding sequences for archaellar or pili-like structures as well as for peptidases involved in processing pre-archaellins or pre-pilins indicating they cannot assemble an archaellum (archaeal flagellum) or type-IV-pili (Fricke et al., 2006; Samuel et al., 2007). Thus, adhesion of cells to the smooth surface of mica plates might also occur via interactions of either the heteropolysaccharide layer surrounding the cells of these two strains or by attachment of unknown cell appendages. Besides, under various stress conditions such as the treatment with human-derived antimicrobial peptides, alterations of the cell wall structure and increased cell aggregation of *Methanosphaera stadtmanae* were observed in an earlier study (Bang et al., 2012). Furthermore, investigations of *Methanobrevibacter smithii* fecal strains as well as of *Methanosphaera stadtmanae* revealed genomic adaptations to the human gut ecosystem such as the production of surface glycans resembling those found in the gut mucosa and a regulated expression of adhesion-like proteins (ALPs) (Fricke et al., 2006; Samuel et al., 2007). The expression of *Methanobrevibacter smithii*’s ALPs was later shown to differ between studied strains and to depend on the existing concentration of formate (Hansen et al., 2011). Since biofilm formation often occurs during strong variations in living conditions such as nutrient limitations (Donlan, 2002; Dongari-Bagtzoglou, 2008), it might also be possible that biofilm formation of methanoarchaeal strains is induced under certain stress conditions involving differential gene expression of ALPs among others. In this respect, it has also been shown that *Methanosarcina mazei* strain S-6 establishes multicellular forms (lamina) under certain stress conditions, which is thought to occur in adaptation to environmental changes (Mayerhofer et al., 1992). Besides, in response to changing culture conditions *Methanosarcina mazei* is able to switch between growth as (sarcina)packages and single cells (Sowers and Gunsalus, 1988). Thus, for *Methanosarcina mazei* it is also likely that it diversifies its cellular growth under static growth conditions in order to form a biofilm.

In summary, the present study demonstrated for the first time that methanoarchaeal strains inhabiting the human gut have the ability to build up biofilms under static conditions. Though focusing on the evaluation of biofilm formation on abiogenic substrates, strong evidence was obtained that *Methanosphaera stadtmanae* and *Methanobrevibacter smithii* might occur as an additional microbial part of mucosal biofilms in the human gut. This is in agreement with previous studies that demonstrated the interaction of these methanoarchaeal strains with bacterial gut commensals such as *Bacteroides* species (Samuel and Gordon, 2006; Samuel et al., 2007). Microbial communities that occur in biofilms on the mucosal surface are currently thought to be crucially involved in modulating the host’s immune system, since they are closer to the epithelium compared to microorganisms in the lumen (Macfarlane and Dillon, 2007; Macfarlane et al., 2011). More importantly, mucosal biofilms have been shown to be associated with many human infectious diseases that are reviewed in (Dongari-Bagtzoglou, 2008). In particular, the composition and density of mucosa-associated biofilms have been shown to alter in individuals with IBD, hence revealing evidence for an impact of sessile communities to human’s gut diseases (Swidsinski et al., 2005). Regarding to this, increased prevalence of *Methanosphaera stadtmanae* was recently found in patients with IBD (Blais-Lecours et al., 2014). Moreover, we recently demonstrated severe activation of human innate immune responses after exposure to this methanoarchaeal strain, which might implicate its contribution to pathological conditions in the human gut (Bang et al., 2014). Thus, the observation in the present study demonstrating biofilm formation of mucosa-associated methanoarchaeal strains might be important for the influence of *Methanosphaera stadtmanae* and *Methanobrevibacter smithii* on the immunomodulation within the human gut that needs to be further elucidated.

## AUTHOR CONTRIBUTIONS

Corinna Bang, Claudia Ehlers, Alvaro Orell, Marlene Spinner, Sonja-Verena Albers and Ruth A. Schmitz designed the research, Corinna Bang, Claudia Ehlers, Alvaro Orell, Daniela Prasse, and Marlene Spinner performed the research, Corinna Bang, Claudia Ehlers, Alvaro Orell, Marlene Spinner, Stanislav N. Gorb, Sonja-Verena Albers and Ruth A. Schmitz analyzed the data, and Corinna Bang, Claudia Ehlers, Alvaro Orell, Marlene Spinner, Sonja-Verena Albers, and Ruth A. Schmitz wrote the paper.

## Conflict of Interest Statement

The authors declare that the research was conducted in the absence of any commercial or financial relationships that could be construed as a potential conflict of interest.
